# Acidity promotes degradation of multi-species environmental DNA in lotic mesocosms

**DOI:** 10.1038/s42003-017-0005-3

**Published:** 2018-01-22

**Authors:** Mathew Seymour, Isabelle Durance, Bernard J. Cosby, Emma Ransom-Jones, Kristy Deiner, Steve J. Ormerod, John K. Colbourne, Gregory Wilgar, Gary R. Carvalho, Mark de Bruyn, François Edwards, Bridget A. Emmett, Holly M. Bik, Simon Creer

**Affiliations:** 1 Molecular Ecology and Fisheries Genetics Laboratory, School of Biological Sciences, Bangor University, Bangor, Gwynedd LL57 2UW UK; 2 Water Research Institute and Cardiff School of Biosciences, Cardiff, CF10 3AX UK; 3 NERC Centre for Ecology & Hydrology, Environment Centre Wales, Deiniol Road, Bangor, Gwynedd LL57 2UW UK; 4 School of Biological Sciences and Natural Resources and Geography, Bangor University, Deiniol Road, Bangor, Gwynedd LL57 2UW UK; 5 Department of Ecology and Evolutionary Biology, Cornell University, Ithaca, NY 14850 USA; 6 School of Biosciences, University of Birmingham, Edgbaston, B15 2TT UK; 7 School of Life and Environmental Sciences, The University of Sydney, Sydney, NSW 2006 Australia; 8 Centre for Ecology & Hydrology, Wallingford, OX10 8BB UK; 9 Department of Nematology, University of California–Riverside, Riverside, CA 92521 USA

## Abstract

Accurate quantification of biodiversity is fundamental to understanding ecosystem function and for environmental assessment. Molecular methods using environmental DNA (eDNA) offer a non-invasive, rapid, and cost-effective alternative to traditional biodiversity assessments, which require high levels of expertise. While eDNA analyses are increasingly being utilized, there remains considerable uncertainty regarding the dynamics of multispecies eDNA, especially in variable systems such as rivers. Here, we utilize four sets of upland stream mesocosms, across an acid–base gradient, to assess the temporal and environmental degradation of multispecies eDNA. Sampling included water column and biofilm sampling over time with eDNA quantified using qPCR. Our findings show that the persistence of lotic multispecies eDNA, sampled from water and biofilm, decays to non-detectable levels within 2 days and that acidic environments accelerate the degradation process. Collectively, the results provide the basis for a predictive framework for the relationship between lotic eDNA degradation dynamics in spatio-temporally dynamic river ecosystems.

## Abstract (web summary)

Mathew Seymour *et al.* investigate the persistence of environmental DNA (eDNA) in river systems in environments of varying pH. Using four sets of upland stream mesocosms, they find that eDNA degrades to non-detectable levels within two days and this degradation is accelerated in acidic environments.

## Introduction

Accurate biodiversity assessment requires reliable species detection and quantification and is essential for furthering our understanding of the natural world and for implementing effective management practices. Traditional biodiversity assessment methods are increasingly being supplemented, or even replaced, with faster and more accurate molecular environmental DNA (eDNA)-based approaches. eDNA is obtained by sampling and directly extracting DNA from natural systems, such as river water, without directly isolating the target organism(s); eDNA is thus freely distributed and originates from sources such as decaying tissue, feces, shed exoskeletons, skin, as well as other bodily excretions^1^. The successful application of eDNA-based approaches in ecology is relatively recent, but several key eDNA studies have already had a major impact on the management of invasive and endangered species^2, 3^, and on biodiversity and environmental assessments^4–7^. However, despite the burgeoning applications of eDNA, there still is limited understanding of the temporal, physical, and chemical factors that influence eDNA persistence dynamics, including eDNA degradation and transport.

Understanding eDNA persistence dynamics is particularly important for ensuring the accuracy and reliability of eDNA biodiversity assessments. Here we define persistence dynamics as the relationship between physical, abiotic, or biotic factors and the degradation and localized detection of eDNA in natural ecosystems. Environmental DNA studies to date have primarily assessed spatially static or semi-static lentic (e.g., pond and lake) or marine environments^6–9^. Particularly, physical hydrological processes, including flow, dilution, and sediment uptake have been shown to influence eDNA detection^10–12^. Lentic eDNA studies have shown reliable analytical species detection in diverse communities^6, 7^, as well as efficient monitoring of rare and low-abundance species^9^. However, the effects of environmental variability among sampling points in relation to findings are largely ignored despite the fact that the persistence of eDNA is directly influenced by the physical and abiotic environment^1^. As is well known to forensic science, tissue and genetic material can persist for extended periods of time in conditions where oxygen and microbial action are reduced or absent, such as DNA extracted from museum specimens or sediment and ice cores^13^. However, DNA can degrade rapidly (i.e., minutes) in aquatic environments due to hydrolysis, oxidation, and microbial activity^14, 15^. The perceived low persistence of DNA in aquatic environments makes the application of aquatic eDNA approaches to biodiversity assessments and environmental management quite attractive, as the short persistence time allows for near real-time monitoring.

Direct tests of eDNA persistence have been limited to single species exclusion experiments in lentic mesocosms^4, 12, 16–20^ or stream cages^21^. While microcosm experiments have shown that increased temperature and lower pH promote eDNA degradation of single species eDNA under control settings^17, 20^, we currently lack an assessment of natural environmental variation on eDNA persistence in the water column across multiple distantly related species. Biotic factors are also expected to influence eDNA persistence in the water column of lotic systems, whereby once eDNA is released, it is expected to settle and accumulate into substrates or biofilms. While higher eDNA concentrations have been found in sediments vs. water samples^10^, the temporal accumulation of eDNA into lotic or lentic substrate has yet to be empirically tested. Overall, understanding how and where detection rates are influenced by environmental factors is paramount for utilizing eDNA methods effectively across systems in order to assimilate knowledge of biodiversity trends.

Despite their ecological and socio-economic importance, lotic systems (i.e., rapidly moving freshwater bodies such as rivers and streams) have rarely been the focus of eDNA investigations. Moreover, the focus of lotic eDNA studies has been on assessing the spatial signal of transporting eDNA, with disparate results suggesting that the eDNA transit distance ranges from meters to kilometers^11, 22–25^. Disparities in these findings likely relates to several physical factors. The transport of a genetic signal will depend on the hydrological dynamics of flow, diffusion/dilution, sinking of the material into the substrate, and subsequent resuspension until the eDNA source becomes degraded beyond the level of capture^10–12^. The range of factors relating to the transit of eDNA will strongly affect our ability to detect biodiversity signals and to date, there have been no studies that assess how environmental factors affect the persistence of lotic eDNA. Consequently, there is a clear need to experimentally assess temporal eDNA dynamics occurring in natural lotic systems.

Here we assess the persistence dynamics of lotic eDNA using a replicated set of semi-natural field experimental streams (i.e., mesocosms) to understand the effects of time and abiotic environmental variation on multispecies eDNA detection. Specifically, we test the effects of a wide range of environmental variables routinely measured for environmental quality, UV, and temperature and address three key knowledge gaps: (1) How does the temporal degradation of eDNA vary across a range of taxonomically disparate species? (2) Which environmental factors can be attributed to static and temporal variation in the eDNA signal? Finally, (3) Does eDNA accumulate in natural stream substrata? Our findings show that multispecies lotic eDNA, derived from water and biofilm, degrades rapidly over time following a negative binomial distribution. Additionally, acidic environments accelerate the rate of lotic eDNA degradation.

## Results

### Environmental variation

**Fig. 1:**
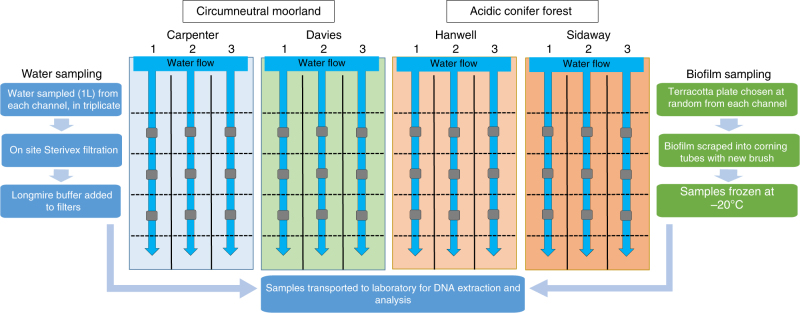
Schematic overview of the study design. The study design includes the sampling workflow for the water and biofilm eDNA sampling. Mesocosms are depicted with their associated names above. The dotted lines represent 1 m channel sections (20 m in total for each channel) in which terracotta tiles (small ceramic tiles) were placed for biofilm accumulation. Background colors (blue, green, orange, red) correspond to the natural acidic gradient of the mesocosms

**Fig. 2:**
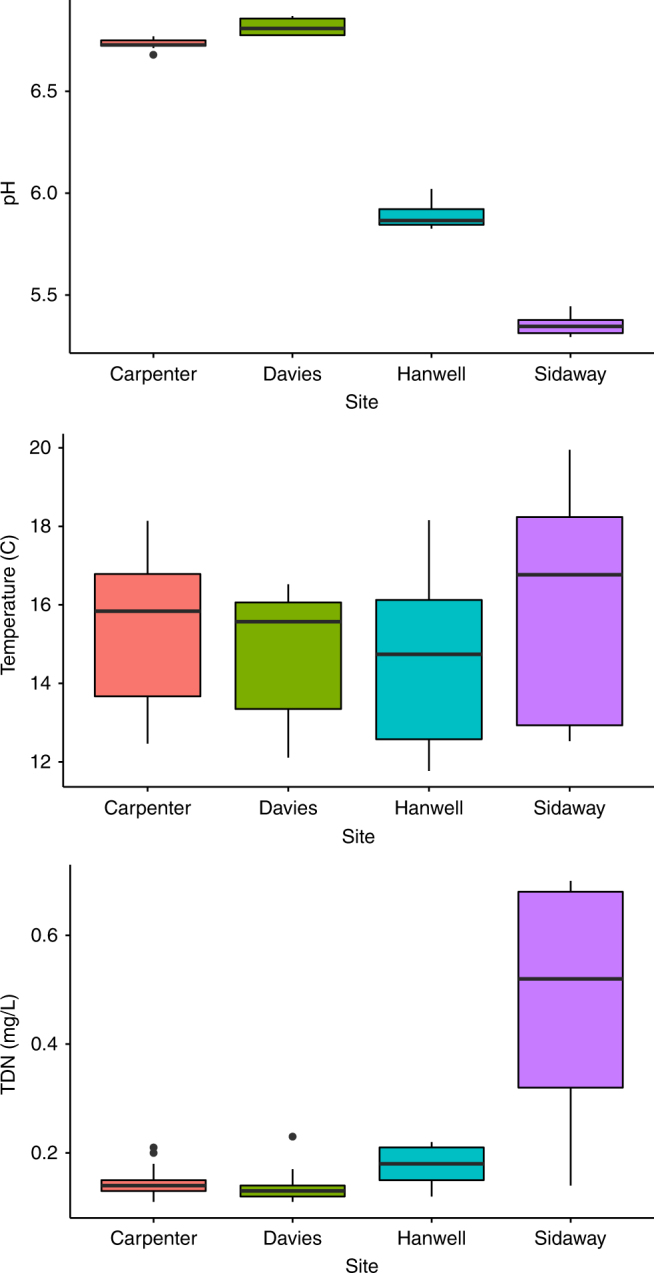
Environmental variation of the experimental flumes. Boxplots showing environmental variation across sites (*x*-axis) for pH (top panel), temperature (middle panel), and total dissolved nitrogen (TDN) (bottom panel). Data shown include daily averages across 3 days with three samples taken per sampling site (one per channel). The upper and lower whiskers show the standard deviation

The experimental sites utilized an established set of mesocosms that were designed specifically to allow experimental lotic comparisons across an environmental gradient present across the Welsh upland, and more generally, representing land uses across the United Kingdom. Each site consisted of four circulating experimental mesocosms, with three channels per mesocosm and with water originating from neighboring streams (Fig. 1). More specifically, mean pH for each mesocosm were typical of the Llyn Brianne catchment^26^ with mean values of 6.73 (±0.01) for Carpenter, 6.82 (±0.04) for Davies (both circumneutral moorland), 5.90 (±0.07) for Hanwell, and 5.35 (±0.05) for Sidaway (both conifer forest). Temperature means were 15.29 °C (±1.80) for Carpenter, 14.72 °C (±1.52) for Davies, 14.47 °C (±1.87) for Hanwell, and 16.16 °C (±2.57) for Sidaway. Mean total dissolved nitrogen was 0.15 mg/L (±0.03) for Carpenter, 0.14 mg/L (±0.03) for Davies, 0.17 mg/L (±0.03) for Hanwell, and 0.49 mg/L (±0.20) for Sidaway (Fig. 2). Additional water chemistry data, measured but not included in the final analyses, are included in the Methods section and Supplementary Material (Supplementary Table 1). For the source eDNA material, we chose ecologically relevant and taxonomically diverse taxa that could either be cultured, or collected to serve as eDNA source material. Thus, *Daphnia magna*, *Ephemera danica*, and *Anguilla anguilla* were selected, thereby facilitating comparisons of eDNA persistence from diverse sources of macroinvertebrates and vertebrates. *Daphnia magna* is a small planktonic crustacean, found commonly in lentic environments across the Northern hemisphere and is routinely utilized in ecological and evolutionary studies. *Ephemera danica* is a species of mayfly commonly found in lakes and rivers across Europe. *Anguilla anguilla* is a critically endangered eel species found in marine and inland waters across Europe and Northern Africa.

### Quantitative PCR

**Fig. 3:**
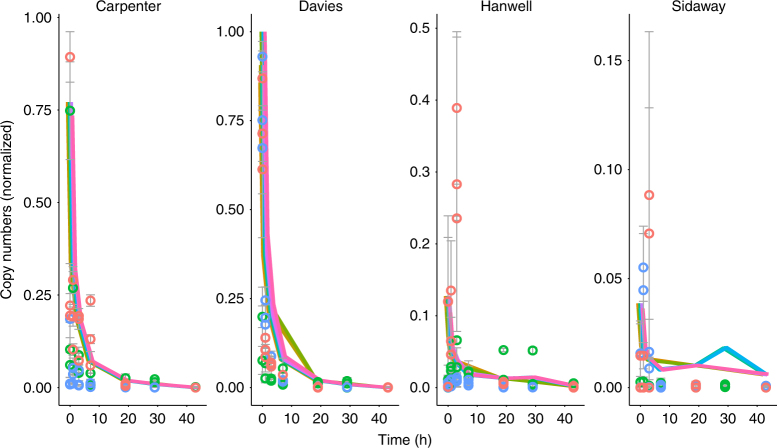
Temporal eDNA dynamics. Results of the qPCR analysis. Quantity (*x*-axis) as normalized copy numbers relative to time (*y*-axis) in hours with each point showing mean quantity values (*n* = 3) for each time point at the respective experimental stream (separate panels). The experiment consisted of 864 data points evenly distributed across three species, four sites, and eight time points with nine samples taken per site per time point (three per channel). Whisker bars show the standard deviation. Lines are the fitted values from a generalized linear mixed effects model. Lines and point data were normalized after fitting the statistical model. Colors represent unique species (*D. magna*, *E. danica*, *A. anguilla*) for each stream replicate (three per stream)

Successful amplification of eDNA from water samples for *D. magna*, *E. danica*, and *A. anguilla* occurred across time points 0, 1, 3, 7, 19, 29, and 43 h, whereas no amplification was observed for all samples at time point −1 (the control sample), where the streams were sampled prior to adding eDNA to the experiment. Additionally, no amplification was evident in the negative PCR controls. Generally, across all species, amplification, calculated as copy numbers, as described in the Methods section, was greatest at time point 0 across all sites (*D. magna*: *x̅* = 18.55 copy numbers ± 34.673, *E. danica*: *x̅* = 56.872 copy numbers ± 95.991, *A. anguilla x̅ *= 2.97 copy numbers ± 3.405) and degraded over time to near 0 copy numbers or null amplification at hour 43 (Fig. 3). While the added sucrose signal decayed over time indicating uptake by the microbial community, the effects of sucrose on DNA quantification was non-significant. Using a mixed effect generalized linear model with a negative binomial error distribution, the variance among groups was ~0 after testing the relation between quantification and time. Therefore, sucrose was not retained as a factor in subsequent analyses. Biofilm eDNA quantification was successful for *E. danica*, but failed for *D. magna* and *A. anguilla*, with lower copy numbers at time point 0 (*x̅* = 2.003 copy numbers ± 3.548), compared to the water-derived eDNA signal, and degrading to near 0 copy numbers at time 43. We assessed whether the lower detectability associated with the biofilm extracts could be due to PCR inhibition by randomly selecting seven samples from time point 0 across the mesocosms and using OneStep PCR Inhibitor Removal Kit (Zymo Research Corp.) prior to rerunning the quantitative PCR (qPCR) with clean and uncleaned samples. Amplification of the cleaned samples did not differ between the cleaned and uncleaned extractions.

**Fig. 4:**
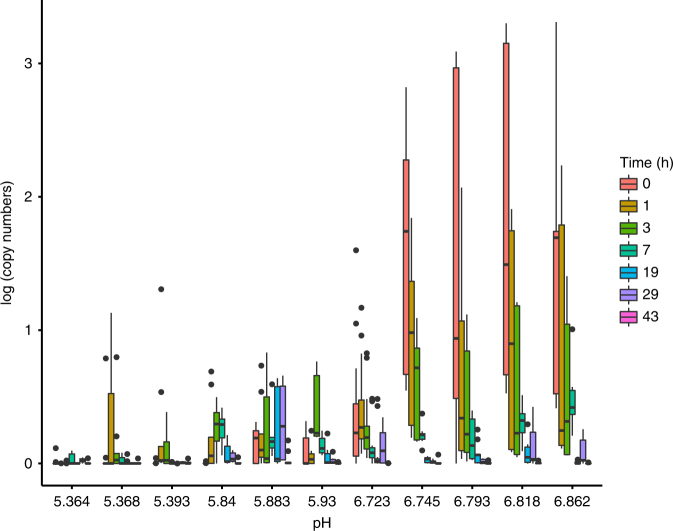
Acidic effects on eDNA detection. Barplot showing eDNA quantification (log copy numbers: *y*-axis) vs. pH (*x*-axis). Each bar depicts the mean quantification value (with accompanying standard deviation) across all samples for a given site/channel, which corresponds to a mean pH value for the given sampling location. The experiment consisted of 864 data points evenly distributed across three species, four sites, and eight time points with nine samples taken per site per time point (three per channel). The different color bars depict different time points including 0, 1, 3, 7, 19, 29, and 43 h from the start of the experiment

We found significant negative effects of time (*P* < 0.001, standard error (SE) = 0.663, slope = −0.100), a significant positive effect of pH (*P* < 0.001, SE = 0.187, slope = 0.926), and a significant negative effect of time × pH (*P* < 0.001, SE = 0.020, slope = −0.092) on water-derived eDNA signal (Table 1; Figs. 3, 4). Random effects of time and species had non-zero standard deviations of 0.554 and 1.048 respectively, indicating their importance to the model. Temperature and total dissolved nitrogen, including their interactions with time, were not significantly related to eDNA quantification and were dropped from the final model. Environmental DNA quantification was typically 1–2 orders of magnitude greater in higher pH (>6) sites compared to lower (<6) pH sites (Fig. 4) shortly after the start of the experiment. Decay rates (proportional loss per hour) derived from the model showed rapid eDNA decay calculated at hour 1 and 3 of the experiment, particularly for the acidic sites Sidaway (0.982 ± 0.001; 0.329 ± 0.001) and Hanwell (0.946 ± 0.005; 0.322 ± 0.001) compared to the circumneutral sites of Carpenter (0.674 ± 0.009; 0.273 ± 0.001) and Davies (0.602 ± 0.030; 0.261 ± 0.005). Biofilm-derived eDNA was not detected in the most acidic mesocosms, with quantification levels roughly 10 times less than those found in the water-derived eDNA. Overall, biofilm-derived eDNA was found to decline significantly over time (*P* < 0.001, SE = 0.008) and was significantly greater at higher pH (*P* < 0.001, SE = 0.184) (Table 2). Decay rates for the biofilm-derived eDNA at the onset of the experiment were much slower in the circumneutral mesocosm, Davies (0.085 ± 0.014; 0.049 ± 0.014) compared to the acidic mesocosm, Hanwell (0.719 ± 0.023; 0.246 ± 0.023).

**Table 1:** eDNA mixed effects model results

Parameter	Estimate	*z*-value	Standard error	*P*-value
*Fixed effects*				
Intercept	−2.389	0.663	−3.602	
Time	−0.099	0.020	−4.863	<0.001
pH	0.926	0.261	3.549	<0.001
Time:pH	−0.092	0.020	−4.503	<0.001
*Random effects*				
	Variance	Standard deviation		
Time	0.307	0.554		
Species	1.097	1.048		

Results of the generalized linear mixed effects model with negative binomial error distribution describing the relationship between quantified copy numbers as the response variable, time, pH, and time × pH as the explanatory variables (fixed effects), and time and species as the random effects. Provided are the values for the estimate, *z*-value, standard error, and *P*-values for the corresponding fixed effects of the model as well as the variance and standard deviation for the random effect of the model

**Table 2:** Biofilm generalized linear model results

Parameter	Estimate	Standard error	*z*-value	*P*-value
Intercept	−0.444	0.115	−3.855	<0.001
Time	−0.036	0.008	−4.574	<0.001
pH	1.318	0.184	7.162	<0.001

Results of the generalized linear model (glm) with negative binomial error distribution describing the relationship between quantified copy numbers derived from biofilm as the response variable, time and pH as the explanatory variables. Provided are the values for the estimate, *z*-value, standard error, and *P*-values for the corresponding parameters of the model

## Discussion

Environmental DNA is predicted to be a powerful source of information for assessing species and community dynamics, as it allows higher spatial and temporal sampling resolution at increased accuracy compared to traditional methods^2, 27–29^. However, for meaningful inferences from natural systems we need to have a fundamental understanding of the processes that govern the persistence and detection of the eDNA signal when exposed to representative environmental variation. Here we present the first experimental assessment, to our knowledge, of eDNA persistence in lotic environments across multiple species under different pH conditions. We found clear indication that environmental conditions interact with temporal dynamics to influence eDNA persistence. Additionally, we show that short-lived eDNA persistence dynamics are similar across species, indicating a general eDNA persistence model, with a negative binomial distribution, that is particularly relevant for large-scale community studies.

Localized eDNA persistence dynamics are largely unknown, but are suspected to be influenced by environmental conditions with laboratory assessments of eDNA decay suggesting pH and high temperatures as key explanatory variables^17, 19^. Conversely, a recent field experiment found that temperature had no effect on seawater-derived *Scomber japonicas* (chub mackerel) eDNA degradation^30^. Here we show that abiotic variation, specifically acidity, decreases eDNA persistence locally and over time. There were no observed effects of nutrient load (e.g., total dissolved nitrogen) or temperature on eDNA degradation rates, but this may be due to the low nutrient levels and relatively homogeneous cooler temperatures, indicative of temperate upland headwater ecosystems. While there has been no assessment of the effects of the abiotic environment on eDNA derived from natural systems, there are some basic laboratory-based understandings with regard to DNA degradation that support our empirical observations. The structure of DNA is very stable under dry, anoxic conditions; with an estimated half life of ~500 years under ideal conditions^31^, but will decay rapidly (minutes) in oxygenated environments, due to effects such as hydrolysis and oxidation^15^. Degradation of DNA is particularly likely when positively charged enzymes, indicative of acidic conditions (i.e., low pH), are present^14^. The finding of decreased eDNA persistence with decreasing pH and temporal degradation is further supported by a single species eDNA-based laboratory study^17^, whereby proportional detection of *Lithobates catesbeianus* eDNA was shown to be lower at pH 4 compared to pH 7; however, degradation comparisons between pH 7 and 10 were non-significant. Moreover, DNA is traditionally preserved in alkaline buffers (e.g., Tris, EDTA buffer, pH 9), and will degrade if left in water due to acid hydrolysis, particularly below pH 7.5^15^.

Temporal persistence of eDNA has thus far been experimentally assessed for individual or closely related species^11, 17, 18, 32^, with reported persistence times ranging from hours to months. In the Llyn Brianne mesocosms, we observed lotic eDNA persistence over 43 h for three taxonomically distant species, which validates previous findings. However, a majority (>90%) of the eDNA signal, across all mesocosms, was lost within the first 3 h of the experiment and within the first hour for the more acidic environments. Nevertheless, the novel observation here was that the prevailing environmental conditions affected the decay dynamics of the disparate forms of multispecies lotic eDNA in a concerted fashion. Although intuitive, harmonized degradation of disparate forms of eDNA suggest that aquatic eDNA is likely derived from the same biological material (e.g., cellular matter)^33^. Regarding the variance between different times of recorded eDNA persistence, differences in overall temporal persistence between this study and previous studies are likely attributed to source eDNA concentrations or differences in experimental design such as local environmental or mesocosm environmental factors. For example, Jerde et al.^23^ assessed eDNA localized persistence in shallow stream beds and found that eDNA was transported out of the system in minutes by flowing water. Likewise, Wilcox et al.^21^ determined that 50% of *Salvelinus fontinalis* produced eDNA was lost within 100 m of the source (i.e., minutes). Conversely, Strickler et al.^17^ showed that lentic eDNA persisted up to 60 days in experimental mesocosms that harbored roughly similar eDNA concentrations as our experiment. Additionally, studies assessing eDNA detection dynamics in natural environments suggest that detection is limited to <1 month in static lentic systems^4^, and at least 24 h across lotic systems^24^. Overall, the short time persistence found in this study, particularly the rate of decay in the acidic environments, is similar to previous findings looking at lotic eDNA persistence in relation to hydrological dynamics^12, 21^.

Lotic eDNA studies are rare, despite the fact that lotic systems are a substantial source of biodiversity information and harbor a disproportionately high amount of Earth’s biodiversity (>6%) compared to their low surface coverage (0.8%)^34^. Additionally, the dendritic interconnected network structure of lotic systems allows for a single river network to encompass a large geographical area, environmental habitats, and diverse species groups^35, 36^. According to our empirical data here, eDNA from sites across a river network will be transported downstream, potentially allowing ecologists and managers to utilize eDNA assessed from downstream confluence sites to infer biodiversity and community dynamics across a large geographical range and set of environmental conditions^24^. Here we demonstrate qPCR detectable eDNA persistence of 43 h, which corresponds to roughly 35 km in rivers with a flow rate of ~2 m/s, which constitutes an average flow rate in natural rivers. However, other studies show that the eDNA signal will be undetectable downstream from the eDNA source due to dilution by large tributaries at the point of the confluence^11^. However, if effects of dilution by tributaries are limited within a river network, the eDNA can be traceable for over 12 km from the eDNA source^24, 37^. Here, we did not include the effects of dilution, as headwater streams are characteristically not influenced by dilution from neighboring streams, although there may be some effect of groundwater flows. Nevertheless, it is essential to consider all factors associated with the transport of eDNA as it moves through different environments as environmental heterogeneity will directly impact the ability to capture the eDNA signal^1^. A potential caveat of the persistence of eDNA is the large spatial heterogeneity possibly associated with sampling eDNA, particularly in riverine environments. While some applications may benefit from catchment wide assessments, efforts to characterize localized diversity will require alternative methodologies^38^. One potential alternative would be to utilize primers targeting longer sequence fragments, which have been shown to degrade faster compared to shorter fragments, thereby likely of more local origin^9, 39^.

The fate of eDNA is largely unknown, but is closely linked with persistence. Aside from chemical decomposition of free-floating DNA molecules and liberation of eDNA from the cell matrix, it is suspected that eDNA will settle at the bottom of river beds and become trapped by the biofilm, which in turn will allow microbial organisms to utilize the accumulating eDNA as a food source^1^. Here, we found little support for eDNA accumulation in the biofilm as quantification failed for two of the three experimental species and the quantification of the *E. danica* biofilm eDNA was a magnitude lower compared to the water-derived *E. danica* quantification. Additionally, the sampled area to total flume area were the same order of magnitude for the water (0.13% of the total volume) and biofilm (0.14% of the total volume) samples. This might suggest that the turbidity of the flowing lotic system does not allow measurable eDNA accumulation. No study to our knowledge has previously assessed eDNA accumulation in biofilm, although previous work by Barnes et al.^18^ showed that *Cyprinus carpio* eDNA degradation increased under lower aerobic activity and chlorophyll levels, which suggest biological activity is either counterintuitively assisting eDNA preservation, or that the effect of biological utilization of eDNA may be less fundamental than expected. Another recent study also showed that the localized retention and resuspension of eDNA in lotic systems is influenced by the substrate type of the river channel, whereby finer substrate beds allow for greater *C. carpio* eDNA substrate uptake^12^. The lower accumulation found in our experiment may therefore be due to the coarse substrate hindering absorptions due to negatively charged surface areas or from the utilization of eDNA as a food source by microorganisms in the substrata^15^. While the findings presented here suggest limited to no additional effect of biological activity on eDNA persistence, further assessment should be made in higher nutrient (e.g., available nitrogen or phosphorous) sites.

This study is the first, to our knowledge, to assess the effect of abiotic factors on eDNA detection and degradation across a suite of ecologically relevant, yet taxonomically divergent taxa in near natural, replicated experimental streams. Overall, the results of this study indicate more rapid eDNA degradation in lotic systems, compared to previous lentic studies, likely attributed to variation in the abiotic environment and physical characteristics of flowing water systems. Additionally, we show that eDNA persistence dynamics are consistent across broad taxonomic groups, further cementing eDNA-based approaches as an efficient, robust method for assessing community dynamics. The findings from this study have clear implications for eDNA approaches to measuring biodiversity in flowing waters, highlighting the need to consider environmental variation among sites, and spatial-temporal dynamics, which are paramount for robust ecological and environmental assessments of biodiversity. Spatio-temporal patterns of species detection are likely to be predictable across different species and strongly influenced by environmental variation across different river catchments.

## Methods

### Experimental setup

We utilized four unique experimental stream mesocosms located upstream of the Llyn Brianne Reservoir (UK; 52.132614, −3.752174) in upland Wales (http://www.cardiff.ac.uk/llyn-brianne-observatory). Each of the experimental streams, described in detail in Durance et al.^40^ (Fig. 1), consisted of three circulating channels (20 m×20 cm×20 cm), utilizing cobble (*D*_50_ = 5 cm) for substrate, with an average flow rate of ~2 m/s with water sourced directly from adjacent headwater upland streams. The experimental channels, with corresponding site names in parentheses, included two channels feeding from moorland catchments with circumneutral waters at pH ranging from 6.8 to 7.2 (L6-Carpenter, L7-Davies), and two from conifer forest catchments with acidic waters at pH ranging from 5.3–5.8 (L3-Hanwell, and recently logged L8-Sidaway). The mesocosms at the Llyn Brianne observatory are fed directly from natural streams, with chemical conditions representing the prevailing acid–base gradient in the upper Tywi catchment^41^. Moreover, the environmental variation represented in the experiment is representative of wider conditions across the whole of upland Wales and, more generally, of large areas of upland Britain^42, 43^.

### Environmental DNA sources and addition

eDNA was sourced from a wide range of taxa including *D. magna*, *E. danica*, and *A. anguilla*. Species were selected with the aim to acquire broad phylogenetic diversity, and based on locally available non-invasive species that were naturally occurring in the Llyn Brianne catchment. *Daphnia magna* were cultivated in mesocosms (~200 individuals/L) at Bangor University, which originated from a single clone provided by Birmingham University. *Ephemera danica* were collected near Galsbury, UK and kept in mesocosms (~100 indv/L) at Bangor University 2 weeks prior to the experiment. eDNA-rich water from the *D. magna* and *E. danica* cultures was collected by sieving individuals from the water using a 250 micron sieve into sterilized plastic containers. *Anguilla anguilla* was sourced from the Cynrig Fish Culture unit (Brecon, UK) where *A. anguilla* juveniles (250 indv/L) were kept in 4 L tanks. Prior to collection, the water from the Cynrig Fish Culture Unit was subjected to ultraviolet light due to water treatment protocols.

At each experimental mesocosm, we added 2 L of eDNA-rich water that had held *D. magna*, and *A. anguilla* and 1 L of eDNA for *E. danica*. The reduced volume for *E. danica* was due to higher eDNA concentration in the holding tanks. We quantified eDNA concentrations prior to addition thereof to the experimental systems using a Qubit (2.0) fluorometer (Life Technologies, Carlsbad, USA) for each species resulting in 5.45 ng/μL (5.45E6 ng/L) for *D. magna*, 7.33 ng/μL (7.33E6 ng/L) for *E. danica*, and 1.75 ng/μL (1.75E6 ng/L) for *A. anguilla*. DNA concentrations were then diluted upon addition to the mesocosms by 1:400 for the *D. magna* (18,600 ng/L) and *A. anguila* (4375 ng/L) and 1:800 for the *E. danica* (9162.5 ng/L), which were over five orders of magnitude higher than concentrations found in natural river systems^44, 45^. Starting eDNA concentrations were also quantified using qPCR as described below.

Furthermore, to test the effect of increased microbial activity on eDNA persistence, a synthetic form of dissolved organic carbon sucrose (>99.0% sucrose, Sigma-Aldrich, Dorset, UK) was added to one of the three channels in each of the experiment streams to simulate high-productivity sites.

### Sampling

Water samples were collected, from the water column, over the course of 44 h, including 1 h prior (time point −1) to the addition of eDNA to the systems (negative control), 10 min after adding eDNA to the system (time point 0) and 1, 3, 7,19, 29, and 43 h from initializing the experiment. In addition to the T −1 negative control sampling, we took one negative control sample for each time point that consisted of previously autoclaved water kept in the same sampling containers as the samples, and kept among the sampled material during the experiment. For each sampling time, 1 L water samples were collected, without replacement, using sterilized Nalgene bottles, in triplicate, from each experimental stream channel, resulting in 36 samples per time point (total 252 samples for the experiment). Compared to the total volume of the mesocosm (800 L), each filtered sample constituted 0.13% of the total mesocosm volume. Water samples were filtered on-site using 0.22 µm Sterivex filter units with male and female luer ends (Millipore Corp, Bilerica, USA) and a Geotech peristaltic pump (series II Geotech, Denver, USA). The eDNA was preserved by expelling all water from the filter units, capping the male luer end with a luer screw cap, filling the sterivex unit with Longmires solution^46^ and capping the female luer end. Samples were then transported to Bangor University, kept at 4 °C and the DNA was extracted within 2 weeks.

To investigate whether eDNA was settling and accumulating on the bed of the channels, we took standardized biofilm samples from three of the experimental channels, covering the full environmental variability. Terracotta tiles (15 cm × 15 cm × 5 cm) were added to 1 m interval sections of the flumes 2 weeks prior to the experiment to allow biofilm growth. During each water sampling event, a tile was removed at random, from each of the flumes in the experimental stream and scraped clean into a 50 mL tube, using standard biofilm sampling protocols^47^. Biofilm samples were then stored at −20 °C and shipped to Bangor frozen for subsequent analyses. Compared to the total surface area of the mesocosm (160,800 cm^2^), each biofilm sample (750 cm^2^) constituted 0.47% of the total sampling surface area.

### Water chemistry

Water chemistry measurements were collected daily for aluminium (Al), boron (B), calcium (Ca), iron (Fe), potassium (K), magnesium (Mg), manganese (Mn), sodium (Na), sulfur (S), silicon (Si), total suspended solids (TSS), bromide (Br), chloride (Cl), fluorine (F), ammonium (NH4-N), nitrite nitrogen (NO2-N), nitrate nitrogen (NO3-N), phosphate (PO4-P), total organic nitrogen (TON), non-purgeable organic carbon (NPOC), total dissolved nitrogen, pH, alkalinity (GranAlk), and conductivity (Cond.). Additionally, temperature and light data loggers (model 650MDS, YSI Inc, USA) were placed in each experimental channel with measurements taken every 15 min during the experiment with daily averages used in subsequent analyses (Supplementary Table 1).

### DNA extraction and qPCR analyses

All extractions and qPCR setups were performed in a designated eDNA laboratory at Bangor University, in rooms free of PCR products (i.e., no PCR machines and no prior PCR amplification occurring in the rooms) with positive air flow. The eDNA was extracted from the filters using a modified Qiagen Blood and Tissue DNeasy (Qiagen, Hilden, Germany) extraction method^48, 49^. In short, the Longmire’s solution was first removed by passing the Longmires through the filter membrane. Lysis buffer and proteinase K were then added to the filter, and the filter placed in a hybridization oven to rotate and incubate at 56 °C overnight. Subsequent extraction steps followed the standard Qiagen DNeasy extraction protocol. We extracted DNA from biofilm samples using PowerMax Soil DNA isolation kits (MoBio) according to the manufacturer’s instructions, following a 20 min centrifuge spinning of the samples at high speed to pellet the sample.

Quantification of extracted eDNA from all water and biofilm samples was performed in triplicate via species-specific targeted qPCR assays (Table 3) developed by Primer Design Ltd (Southampton, UK). Each 20 μL reaction contained 1 μL primer/probe mix (300 nM), 10 μL (2X) PrecisionPLUS Mastermix (Primer Design Ltd.), 2 μL DNA, and 7 μL DNAse-free water. Reactions were run on a QuantStudio Flex 6 Real-Time PCR System (Applied Biosystems, USA) with the following protocol: 2 min at 95 °C, followed by 40 cycles of 10 s at 95 °C and 60 s at 60 °C. Each qPCR plate included a five-fold dilution series of the relevant control DNA (*D. magna* 6500 copies/reaction to 0.65 copies/reaction, *E. danica* 4000 copies/reaction to 0.40 copies/reaction, *A. anguilla* 1500 copies/reaction to 0.15 copies/reaction) and no template control in triplicate. For each primer set, mean Ct values generated from the control DNA dilution series were plotted against log gene copy number to generate a standard curve and a linear line of best fit to assess amplification efficiency, *y*-intercept and *R*^2^ value.

**Table 3:** qPCR primer/probe information

Target species	Primer/probe	Primer sequence
*Daphnia magna*	Sense	TCGGAATGATCTCTCATATTATCAGTC
	AntiSense	ACCTAAGACACCAATAGCTAATATAGC
	Probe	TCCCAAAGGCTTCCTTCTTCCCTCTTTCG
*Ephemera danica*	Sense	CTTCCTCCTGCTTTAACACTTCTT
	AntiSense	GGGCGATTCCTGCTGCTAA
	Probe	ACAGTTCAACCTGTTCCTGCTCCTCTTTCT
*Anguila anguila*	Sense	GCAGGTATTTCATCAATTCTAGGG
	AntiSense	GAGTAGTAAAACGGCGGTTACTAA
	Probe	ACCGCCTGCAATTACACAGTACCA

Quantitative PCR sense and antisense primer, and probe sequences for each target species used for this study

### Statistical analyses

All statistical analyses and graphics employed R, version 3.3.1^50^. To assess the relationship between eDNA quantification in relation to time and environmental variation, we fitted a mixed effect generalized linear model with a negative binomial error distribution using quantified eDNA copy numbers as the response variable. Initial explanatory variables included time, pH, total dissolved nitrogen, temperature, and all two-way interactions between pH, total dissolved nitrogen, temperature, and time. Water chemistry explanatory variables were selected based on individual variable distributions, particularly avoiding variables with an overabundance of zero values as they likely result from lower detection limitation and may result in type I errors due to zero-inflation^51^. Additionally, highly correlated variables were reduced using pairwise comparisons to avoid violation of independence among explanatory variables. Explanatory variables were centered, such that their mean = 0, prior to model fitting to avoid unrealistic intercept parameterization. Time and species were included as random effects to account for covariance structure among time points and among species (i.e., starting eDNA concentrations). Models were reduced using backward model selection with Akaike information criterion (AIC) comparisons, such that the final model resulted in time, pH, and time:pH as explanatory factors. The relationship between biofilm-derived eDNA in relation to time and environmental variation was assessed in a similar fashion as the water-derived eDNA, except a simpler generalized linear model with a negative binomial error distribution was fitted, as it was determined that including random effects did not improve the model fits.

### Data availability

All data associated with the study are freely available on Figshare 10.6084/m9.figshare.5509525.

## Electronic supplementary material

Supplementary Information
