# Supplementary material for: Acidity promotes degradation of multi-species environmental DNA in lotic mesocosms
Source: Commun Biol. 2018 Jan 22;1:4. doi: 10.1038/s42003-017-0005-3 (PMC6123786; doi:10.1038/s42003-017-0005-3)
Supplement: Supplementary file 1 — Supplementary Information [file 42003_2017_5_MOESM1_ESM.pdf]

Supplementary Table 1. Environmental variables measured during the experiment (Variable). With corresponding means and standard deviations (SD) for each Site (Carpenter, Davies, Hanwell, Sidaway).

		Carpenter		Davies		Hanwell		Sidaway	
Variable	Units	Mean	SD	Mean	SD	Mean	SD	Mean	SD
Aluminum	mg/L	0.06	0.01	0.06	0.01	0.13	0.01	0.28	0.11
Boron	ppb	6.03	0.52	4.45	0.20	6.07	0.19	7.94	1.80
Calcium	mg/L	2.33	0.19	3.39	0.16	1.51	0.04	1.23	0.05
Iron	mg/L	0.15	0.02	0.11	0.03	0.22	0.03	0.64	0.75
Potassium	mg/L	0.08	0.03	0.09	0.02	0.08	0.01	0.56	0.05
Magnesium	mg/L	1.43	0.12	1.54	0.06	0.67	0.03	0.79	0.05
Manganese	mg/L	0.01	0.01	0.00	0.00	0.00	0.00	0.09	0.05
Sodium	mg/L	2.87	0.25	3.83	0.69	4.75	0.10	5.23	0.32
Sulfur	mg/L	1.11	0.32	1.58	0.09	1.69	0.03	1.54	0.33
Silicon	mg/L	0.97	0.17	0.65	0.34	1.21	0.12	1.12	0.30
Total Suspended Solids	mg/L	6.11	3.28	1.03	0.38	1.71	0.56	10.84	7.74
Bromide	mg/L	0.02	0.00	0.01	0.00	0.03	0.00	0.04	0.01
Chloride	mg/L	4.51	0.19	4.66	0.09	6.94	0.14	8.50	0.39
Fluorine	mg/L	0.04	0.00	0.05	0.00	0.02	0.00	0.04	0.00
Ammonium	mg/L	0.00	0.00	0.01	0.01	0.01	0.01	0.01	0.02
Nitrite nitrogen	mg/L	0.01	0.00	0.01	0.00	0.01	0.00	0.01	0.00
Nitrate nitrogen	mg/L	0.04	0.03	0.02	0.03	0.07	0.05	0.27	0.22
Phosphate	mg/L	0.00	0.00	0.00	0.00	0.00	0.00	0.00	0.00
Total Organic Nitrogen	mg/L	0.00	0.00	0.00	0.01	0.07	0.04	0.29	0.26
Non-purgeable organic carbon	mg/L	9.62	9.08	9.67	9.16	10.95	9.02	9.93	6.58
Total dissolved nitrogen	mg/L	0.15	0.02	0.14	0.03	0.18	0.03	0.49	0.20
pH		6.73	0.03	6.82	0.04	5.90	0.07	5.35	0.05
Alkalinity	ueq/L	160.31	9.11	215.43	11.49	58.25	50.59	13.93	1.27
Conductivity	us/cm	37.72	3.52	46.55	1.82	41.80	3.76	46.20	1.41
Temperature	C°	15.29	1.80	14.72	1.52	14.47	1.87	16.16	2.57
Luminosity	cd/m^2	31750	16512	22309	10948	17425	12616	19068	12605
